# Biosynthesis of Silver Nanoparticles via *Medusomyces gisevii* Fermentation with *Origanum vulgare* L. Extract: Antimicrobial Properties, Antioxidant Properties, and Phytochemical Analysis

**DOI:** 10.3390/molecules30081706

**Published:** 2025-04-10

**Authors:** Aiste Balciunaitiene, Syeda Hijab Zehra, Mindaugas Liaudanskas, Vaidotas Zvikas, Jonas Viskelis, Yannick Belo Nuapia, Arturas Siukscius, Pradeep Kumar Singh, Valdimaras Janulis, Pranas Viskelis

**Affiliations:** 1 Institute of Horticulture, Lithuanian Research Centre for Agriculture and Forestry, 54333 Babtai, Lithuania; hijab.zehra@lammc.lt (S.H.Z.); jonas.viskelis@lammc.lt (J.V.); pranas.viskelis@lammc.lt (P.V.); 2 Department of Pharmacognosy, Faculty of Pharmacy, Lithuanian University of Health Sciences, 50166 Kaunas, Lithuania; mindaugas.liaudanskas@lsmu.lt (M.L.); valdimaras.janulis@lsmu.lt (V.J.); 3 Institute of Pharmaceutical Technologies, Faculty of Pharmacy, Lithuanian University of Health Sciences, 50166 Kaunas, Lithuania; vaidotas.zvikas@lsmu.lt; 4 Research Institute of Natural and Technological Sciences, Vytautas Magnus University, 40444 Kaunas, Lithuania; 5 Pharmacy Department, School of Healthcare Sciences, University of Limpopo Campus, Polokwane 0727, South Africa; yannick.nuapia@ul.ac.za; 6 Veterinary Academy, Institute of Animal Husbandry, Lithuanian University of Health Sciences, 50166 Kaunas, Lithuania; arturas.siukscius@lsmu.lt; 7 Department of Mechanical Engineering, Institute of Engineering and Technology, GLA University, PO Chaumuhan, Mathura 281406, India; pradeep.kumar@gla.ac.in

**Keywords:** green synthesis, *Origanum vulgare* L., silver nanoparticles, fermentation, phytochemical analysis, antibacterial activity, antioxidant activity

## Abstract

Silver nanoparticles belong to a highly versatile group of nanomaterials with an appealing range of potential applications. In the realm of antimicrobial and antioxidant application, silver nanoparticles (AgNPs) exhibit auspicious capabilities. This research, for the very first time, endeavors to carry out biosynthesis of AgNPs coupled with fermentation using *Medusomyces gisevii* and *Origanum vulgare* L. (*O. vulgare*) plant species. Fermentation (F) via *Medusomyces gisevii* is responsible for chemical, physical, biological, and electrochemical processes. During in vitro study of antioxidant activity, fermented *O. vulgare* herb extract showed strong reductive activity as evaluated by the cupric reducing antioxidant capacity (CUPRAC), ferric reducing antioxidant power (FRAP), and 2,2′-azino-bis (3-ethylbenzothiazoline-6-sulfonic acid) (ABTS•+) assay, with a value of 1.45 ± 0.048 mmol TE/g, 0.95 ± 0.04 mmol TE/g, and 0.59 ± 0.023 mmol TE/g, respectively. The highest antimicrobial activity was shown by *Staphylococcus aureus* in the inhibition zone, with values of 1.40 ± 0.12 mm of OrV and of 10.30 ± 0.04 mm and 11.54 ± 0.10 mm for OrV-AgNPs and OrV-F-AgNPs, respectively. Analysis of phenolic compounds revealed that the highest total amount of the apigenin, 87.78 µg/g, was detected in OrV-F-AgNPs and the lowest amount, 16.56 µg/g, in OrV-AgNPs. Moreover, in OrV-F-AgNPs, the collective amount of proanthocyanidins, hydroxycinnamic, and flavonoids was prominently high in all cases, i.e., 145.00 ± 0.02 mg EE/g DW, 2.86 ± 0.01 mg CAE/g DW, and 0.55 ± 0.01 mg RE/g DW, respectively, as compared to the original extract (102.1 ± 0.03 mg EE/g DW, 2.78 ± 0.02 mg CAE/g DW, and 0.47 ± 0.01 mg RE/g DW, respectively). During the characterization of biosynthesized nanoparticles by scanning electron microscopy (SEM), AgNPs demonstrated a uniform spherical shape with even distribution. The sample’s elemental composition was confirmed with a signal of 3.2 keV using energy-dispersive X-ray spectroscopy (EDS) analysis. Transmission electron microscopy (TEM) analysis showed silver nanoparticles that were round and spherical in shape in both stacked and congested form, with a size range of less than 30 nm. Thus, this green and sustainable synthesis of AgNPs, a blend of *Medusomyces gisevii* and *O. vulgare* herbal extract, has adequate potential for increased antimicrobial and antioxidant activity.

## 1. Introduction

Nanotechnology is a greatly advanced and diverse field that characterized by multidirectional applications in the twentieth century [1,2]. Using nanotechnology, the functionality of materials can be greatly enhanced, in terms of both their economic and commercial value [3]. Green synthesis of nanoparticles is relatively cheap, non-hazardous, produces less waste, has the lowest production price, and is easy to implement on an industrial scale [4,5,6,7]. For example, a study shows that synthesis of silver nanoparticles in large quantities can be carried out by using the ball milling method [8]. Prominent research articles are available in the literature explaining that different forms of NPs are photochromic polymer NPs [9], core-shell NPs [10], inorganic NPs, CuNPs, AgNPs, PdNPs [11], AuNPs [12], SiNPs [13], NiNPs [14], and PtNPs; however, others are metal dioxide and metal oxide NPs, including CuO [14], FeO [15], ZnO, MgO [16], CeO_2_ [17], TiO_2_, and ZrO_2_ [18]. These nanoparticles (NPs) possess significant applications and unique properties, and they can be synthesized using traditional and unconventional methods. Effective NP formation can be carried out in a renewable or ecologically safe manner using safe capping and reducing agents. Various biomolecules, including enzymes, biodegradable polymers, vitamins, microbes, plant waste, and yeasts, can be used as active agents in the green synthesis of NPs [19,20]. Owing to the presence of green reagents, such as ascorbic acids, amides, terpenoids, ketones, carboxylic acid, phenols, and aldehydes, present in nanomaterials derived from plants, NPs have broad applications [21]. Silver nanoparticles (AgNPs) have efficient antimicrobial activity, as they interact strongly with low concentrations of microorganisms. As silver ions are released in pathogens, several mechanisms are initiated, such as denaturation of protein due to attachments with functional groups, hindering the respiratory chain, cell membrane disruption, and clogging up deoxyribonucleic acid (DNA) replication. Therefore, AgNPs present strong antimicrobial activity against Gram-positive and Gram-negative bacterial strains [22]. The mechanism of reactive oxygen species (ROS) is of great importance for the degradation of pathogenic bacteria. ROS are basically the most reactive and active molecules that contain superoxide anion (O_2_^•−^), hydroxyl radicals (^•^OH), molecular oxygen (O_2_), and hydrogen peroxide (H_2_O_2_). They can carry out antimicrobial actions to suppress a wide variety of pathogens by oxidative stress induction. The imbalance of ROS allows them to act between the defense system of antioxidants to detoxify ROS. Lipids, proteins, DNA, and cellular macromolecules may be damaged by oxidative stress from ROS [23]. M Banerjee reported that iodine combined with a nanocomposite possessed ROS with oxidative stress inside a bacterial cytoplasm, causing cell death [24].

Studies reported that worldwide fatalities due to bacterial antimicrobial resistance rose from 1.27 to 4.95 million in the past 30 years [25]. A study by Balciunaitiene, A, mentioned that around 700,000 deaths occur each year due to antibiotic resistance; by 2050, this number could increase to as many as 10 million [26]. The European Centre for Disease Prevention and Control (ECDC) and US Centers for Disease Control and Prevention (CDC) reported around 0.67 and 2.8 million antibiotic infections and 33,000 and 35,000 deaths each year in Europe and the US, respectively.

The synthesis methods for AgNPs can be chosen from among microwave, biological, photochemical, chemical, physical, and microemulsion methods [26,27,28,29]. Physical methods are not economical, and chemical methods involve using hazardous reducing agents like hydrazinium chloride and many others [30,31,32]. Thus, cutting-edge technology has shifted towards biological synthesis, which is economical and eco-friendly and provides a high yield in green synthesis [33]. However, in biological synthesis, reducing agents are microorganisms [34,35] and parts of plants. Medicine and biology use AgNPs for antimicrobial activity, as they have a powerful biocidal effect on microorganisms [36]. In the past, they have been used to cure infections and ailments. Nowadays, they have applications in coatings, wound dressing, clothing, food containers, etc. [37]. Even though microorganisms can be used for AgNPs’ synthesis, various plants have been used for this owing to their stabilization and plant phytochemicals. Possibly, the antioxidant properties of AgNPs are explained by different factors: flavonoids, vitamin E (alfa tocopherol), vitamin C (ascorbic acid), vitamin A (beta-carotene), and various phenolic compounds [38,39]. Plant extracts have antioxidant and antimicrobial properties, which have received tremendous attention in scientific research.

A promising and research-approved natural antioxidant is oregano. Oregano, also known as “Origanum Vulgare Lamiaceae”, is a perennial sub-shrub with an aroma, and it is distributed in North Africa, Eurasia, and Balkan countries [40]. This plant species vacillates both in its chemical composition and morphology. Considering its biological composition, oregano is placed at the top of the list of prominent species in Europe [41]. Based on classical known taxonomy, oregano has six subspecies. Among them, *O. vulgare* subsp. *hirtum*, well known as Greek oregano, is grown worldwide and considered the most valuable. Oils of oregano have high antioxidant properties, owing to the presence of phenolic components such as carvacrol and thymol. Oregano has wide applications in medicine to cure human diseases, including coughs, skin, wounds, and gastrointestinal problems [42]. Because of their high carvacrol and thymol content, oregano oils have high antibacterial and antioxidant properties in the bread, cheese, and meat industries [43,44]. These properties result from factors such as the plant’s geographical location, genotype, environment, developmental stage, cultural practices, and cultivation season and from the specific plant part selected for usage.

The synthesis of oregano extract (OE) is a fascinating process that can be achieved using various methods, each resulting in a unique phytochemical composition. The aim of our study, in terms of the role of green nanotechnology, is to develop a low-cost and environmentally friendly way to synthesize silver nanoparticles using *oregano* extract to increase the antioxidant and antimicrobial activity of the extract.

## 2. Results and Discussion

### 2.1. Determination of Phytochemical Composition and Antioxidant Activity

Phenolic compounds are specialized metabolites found in plants that play crucial roles in various physiological processes, contributing significantly to plant development and growth. These compounds are involved in structural functions, acting as part of the plant cell wall, and providing mechanical support. Regarding this aspect, it is essential to determine the quantitative composition and variability in the qualitative and quantitative composition of phenolic compounds in raw plant materials. The following flavanols have been identified: quercetin, isorhamnetin and their glycosides, and luteolin-7-O-glucoside. Galangin carries out many biological activities, including antimicrobial, anti-inflammatory, antiobesogenic, antiviral, and antioxidant activities. However, the anti-tumor mechanism of galangin remains unclear [45].

The highest total amount of apigenin, 87.78 µg/g, was detected in OrV-F-AgNPs, and the lowest amount, 16.56 µg/g, was detected in OrV-AgNPs. Rutin, also known as rutinoside or vitamin P, is a polyphenolic bioflavonoid widely found in plants. Rutin has been adapted for variable nanocarriers using different approaches to improve its therapeutic efficacy and storage stability. The highest content of rutin (69.89 µg/g) was found in OrV-AgNPs; the lowest content was found in the pure extract (14.22 µg/g). The highest total amount of identified and quantitatively evaluated compounds (4323.72 µg/g) was found in pure *O. vulgare* herb extract, and the lowest (753.68 µg/g) was found in OrV-AgNPs.

UV-Vis spectrophotometric evaluation of the total composition of phenolic compounds in *O*. *vulgare* herb extracts, OrV-AgNPs, and OrV-F-AgNPs was performed. The results obtained are summarized and presented in Table 1. The highest content of phenolic compounds was found in fermented OrV-AgNPs. In contrast, a lower amount (18%) was detected in the pure *O. vulgare* herb extract. The lowest number of phenolic compounds was found in OrV-AgNPs composition, equal to 206.00 ± 0.59 mg GAE/g. The results of the phytochemical composition obtained for *O. vulgare* herb extracts frequently differ among researchers [46,47]. These differences in phenolic compounds in the same plant can be explained by the fact that other factors not studied may strongly affect the qualitative and quantitative content of various plants’ generative and vegetative organs. For example, the geographic region and the soil’s geochemical composition vary significantly even when analyzing relatively small areas. In addition, climate, meteorology, plant cultivation, raw material storage conditions, and foliar fertilization can vary significantly. All these factors have a direct influence on the number of phenolic compounds. Further, from the data presented, after the synthesis of silver nanoparticles, the number of phenolic compounds decreased to 206.00 ± 0.59 mg GAE/g (~2%). This decrease in phenolic compounds can be explained by the fact that phenolic compounds act as reducers, anticoagulants, and stabilizers. It is theorized that the donating potential of phenols facilitates the formation of metal nanoparticles by the bioreduction of Ag^+^ to Ag^0^ and further stabilization of nanoparticles [48].

The research methodology employed in this study to evaluate the antioxidant activity of plant extracts, a crucial biological effect intricately linked to antibacterial, anti-inflammatory, antiviral, and other biological effects, was comprehensive. Results of the phytochemical analysis are presented in Table 2. To ensure the most accurate evaluation of the antioxidant activity of the research objects, we conducted in vitro studies of antiradical and reductive activity. This involved the use of the reductive FRAP and CUPRAC assays, as well as the ABTS and CUPRAC radical scavenging assays [49,50]. It is worth noting that, based on our analysis of the scientific literature, it is recommended to employ several different methods to assess the antioxidant activity of herbal extracts [51].

Many researchers have reported the antioxidant activity of *O. vulgare* herb sample extracts [52,53]. Table 3 shows the antioxidant activity in vitro of the studied *O*. *vulgare* herb extracts. The pure aqueous extract of *O. vulgare* herb exhibits antioxidant properties based on all tested methods. The *O*. *vulgare* herb extract also showed stronger reductive activity as evaluated by the CUPRAC assay. The antioxidant activity observed with the extracts of *O. vulgare* herb is probably due to phenolic compounds, which are well known for their antioxidant capacity [54,55].

Further, the antioxidant activity of pure *O. vulgare* herb extract and biosynthesized green AgNPs were compared (Table 4). Data obtained by ABTS, FRAP, and CUPRAC assays show that biosynthesized AgNPs possess slightly lower reducing and antioxidant potential than plant extracts. Similar observations were obtained using metal nanoparticles in other plants [56].

### 2.2. Scanning Electron Microscopy (SEM) Analysis

**Figure 1:**
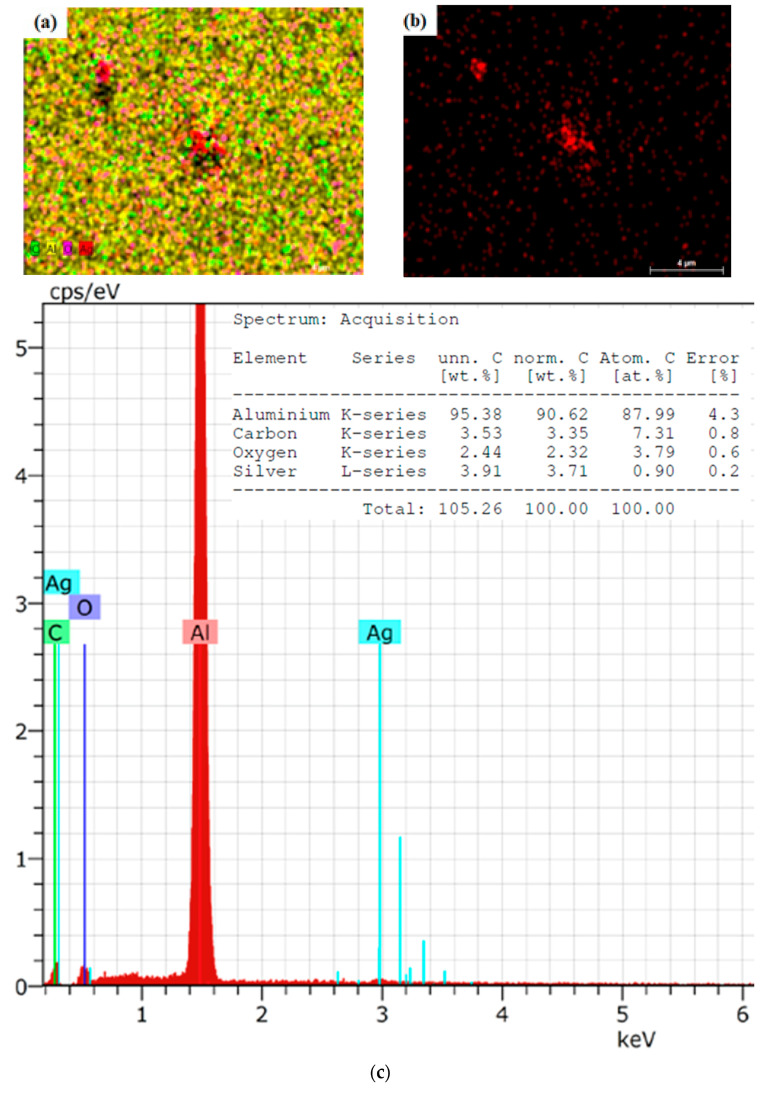
(**a**) SEM image of OrV-F-AgNPs MAG 5000× (**b**) SEM image presenting only AgNPs MAG 5000× (**c**) Elemental analysis of sample.

SEM analysis confirmed the presence of elements like aluminum, carbon, oxygen, and silver. An approximate particle size of less than 50 nm was observed in the images, confirming the sample’s good quality (Figure 1). SEM micrographs of fermented oregano revealed evenly distributed surfaces of nanoparticles unaccompanied by any irregularities and holes. Figure 1b shows the presence of silver only, and it is much more visible with a small nanoparticle size. Figure 1c illustrates the strong signal for silver on the energy of about 3 keV.

**Figure 2:**
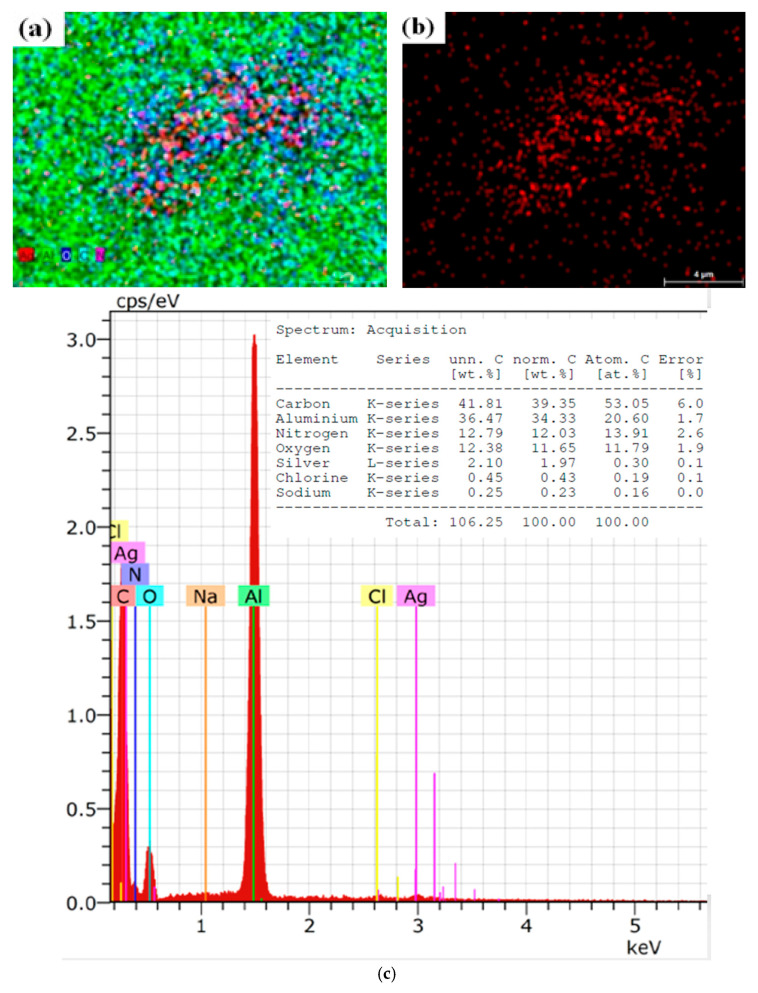
(**a**) SEM image of OrV-AgNPs, MAG 5000× (**b**) SEM image presenting only AgNPs MAG 5000× (**c**) Elemental analysis of sample.

On the contrary, Figure 2 presents the elemental and morphological composition of OrV-AgNPs. The SEM results presented in Figure 2a,b show clear scattered patterns of elements like aluminum, nitrogen, carbon, silver, and others [57]. This is the pattern without any fermentation. Figure 2c is of the EDX spectra, showing the first significant peak of aluminum followed by silver, nitrogen, and others.

### 2.3. Transmission Electron Microscopy (TEM) Analysis

**Figure 3:**
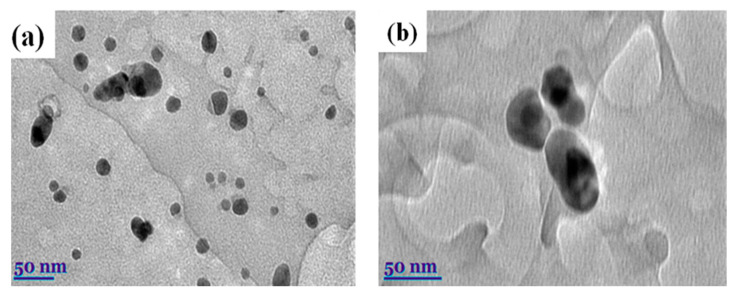
(**a**) TEM micrographs of AgNPs were obtained using *Origanum vulgare* L. (**b**) Fermented extracts of OrV-AgNPs.

Transmission electron microscopy (TEM) is the most powerful approach to provide exhaustive confirmation related to the shape and size of NPs. A Tecnai G2 F20 X-TWIN (FEI, Hillsboro, OR, USA) was used to study these nanoparticles’ structure. An amount of 20–200 kV of voltage was applied using Schottky-type electron emission and a resolution of 0.8–1.0 nm. For visualization, an 11 MPix ORIUS SC1000B camera was used (Gatan Inc., Pleaston, CA, USA). Several different spots were chosen to gain better visualization of the shape of the nanoparticles; then, images were added in the article. Using the TEM analysis technique, the size and shape of synthesized AgNPs were determined (Figure 3). The first image below, Figure 3a, shows silver nanoparticles’ round and spherical shape. Their size was less than 30 nanometers [57].

**Figure 4:**
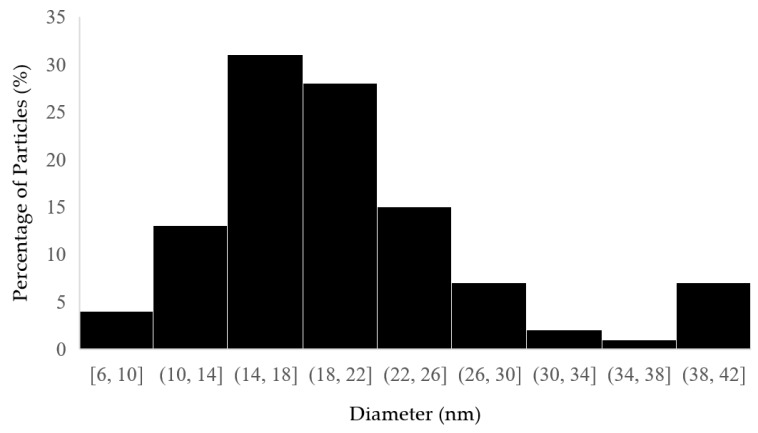
Histogram of distribution size of *Origanum vulgare* L.

**Figure 5:**
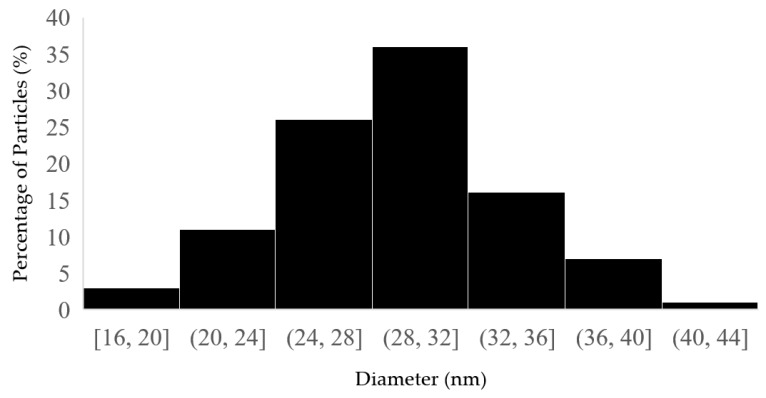
Histogram of distribution size of fermented extracts of OrV-AgNPs.

**Figure 6:**
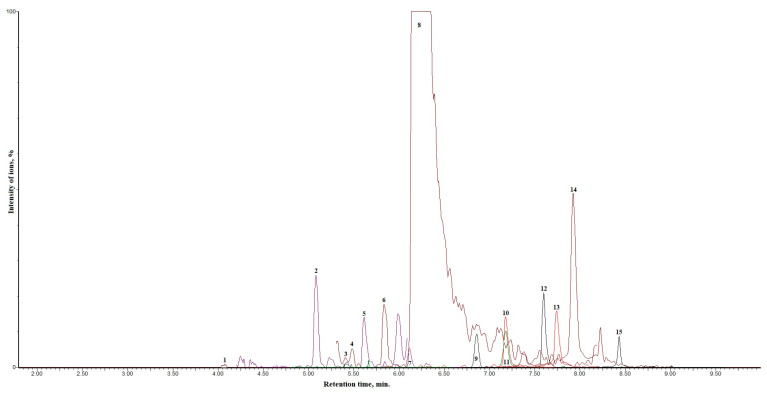
Chromatogram of oregano extract. The identified analytes are expressed as numbers—1: (blue) chlorogenic acid; 2: (dark blue) *p*-coumaric acid; 3: (purple) rutin; 4: (Dark red) hyperoside; 5: (Purple) luteolin-7-0—glucoside; 6: (dark green) isorhamnetin-3-*O*-rutinoside; 7: (Red) luteolin-4′-*O*—glucoside; 8: (Dark brown) rosmarinic acid; 9: (Dark green) luteolin-7-rutinoside; 10: (Red) luteolin; 11: (Red) quercetin; 12: (Black) naringenin; 13: (Red) apigenin; 14: (Mehroon) isorhamnetin; 15: (Black) galangin.

Approximately 100 nanoparticles from the TEM image were analyzed to measure the distribution and the size of the nanoparticles; this was carried out using the ImageJ software (Version 1.54 d, NIH, Bethesda, MD, USA), and the results are presented in Figure 4 and Figure 5. In some places, congested or stacked NPs were observed, whereas in other places, they were apart. Scattered patterns are generally complex to separate, and hence, Figure 3b shows agglomeration. The size of the nanoparticles ranged between 10 and 50 nanometers. A chromatogram of oregano extract is presented in Figure 6.

### 2.4. Antimicrobial Activity

*O. vulgare* herbs have numerous applications in the food, pharmaceutical, and healthcare industry. The presence of phenolic acids, terpenes, flavonoids with predominant thymol and carvacrol, and phenols makes this herb unique, and it is used for its various antimicrobial, antifungal, and antibacterial activities [58]. The most active and significant component in this plant is carvacrol. This study constitutes and contributes to evaluating the antimicrobial activity of extracts and synthesized nanoparticles, which were examined against Gram-positive and Gram-negative bacterial cultures. The agar diffusion method (Kirby–Bauer) was chosen as a technique for evaluating antimicrobial properties. The results show that biosynthesized AgNPsF particles exhibit higher values than AgNPs by both bacteria. Morphological parameters, like size and shape, are responsible for different antimicrobial activities [59].

*Staphylococcus aureus* and *Pseudomonas aeruginosa* showed the topmost and lowest inhibition against OrV-F-AgNPs, with a value of 11.54 ± 0.10 and 4.02 ± 0.10, respectively (Table 4). Similarly, *Staphylococcus aureus* and *Pseudomonas aeruginosa* showed the highest and lowest inhibition against OrV-AgNPs, with values of 10.30 ± 0.04 and 3.85 ± 0.10, respectively. Accordingly, *ß-streptococcus*, *Staphylococcus epidermidis*, and *Enterococcus faecalis* demonstrated relatively lower yet notable levels of inhibition compared to Staphylococcus aureus. This variation in antimicrobial activity can be attributed to the differences in the structural characteristics of the bacterial cell walls, particularly their thickness.

*Staphylococcus aureus*, a well-known pathogen, possesses a relatively thicker and more robust cell wall, which can provide a more formidable barrier against antimicrobial agents. In contrast, the thinner cell walls of *ß-streptococcus*, *Staphylococcus epidermidis*, and *Enterococcus faecalis* may render them more susceptible to the effects of these agents, resulting in observable inhibition despite their lower overall inhibition levels.

Additionally, the composition of the cell wall plays a crucial role in determining susceptibility. For instance, the presence of specific peptidoglycan layers and other structural components can influence how effectively antimicrobial agents penetrate the cell wall and disrupt cellular functions. Therefore, understanding these variations in cell wall structure is essential for interpreting the differences in inhibition observed among these bacterial species. This knowledge can provide insights into the development of targeted antimicrobial strategies and the selection of effective treatment options for infections caused by these microorganisms [60]. The literature lacks deep and advanced knowledge regarding the antibacterial mode of action for the preparation of silver nanoparticles using fermented *O. vulgare* herb extract. Various hypotheses are present in the literature that explain intercellular organelles’ disruption, oxidative stress, cell wall penetration, and changes in signal transduction [61,62].

## 3. Materials and Methods

### 3.1. Chemicals

Tris(2-pyridyl)-s-triazine) and iron (III) chloride hexahydrate (FeCl_3_ × 6H_2_O) were purchased from Merck (Darmstadt, Germany). Ethanol (96.3% *v*/*v*) was obtained from AB Stumbras (Kaunas, Lithuania). Potassium chloride (KCl) was obtained from Scharlau (Barcelona, Spain). Potassium bisulphate (K_2_S_2_O_8_), neocuproine, and DPPH• (2,2-diphenyl-1-picrylhydrazyl)) were obtained from Alfa Aesar GmbH & Co KG (Karlsruhe, Germany). Sulphuric acid (H_2_SO_4_, 95%) was purchased from Chempur (Piekary Śląskie, Poland. Copper (II) chloride dihydrate (CuCl_2_ × 2H_2_O) was purchased from Roth (Karlsruhe, Germany). Ammonium acetate, ABTS (2,2′-azino-bis(3-ethylbenzothiazoline-6-sulfonic acid), Trolox ((±)-6-Hydroxy-2,5,7,8-tetramethylchromane-2-carboxylic acid), TPTZ (2,4,6-Tris(2-pyridyl)-s-triazine), sodium acetate trihydrate (CH_3_COONa × 3H_2_O), alangin, p-coumaric acid, apigenin, naringenin, luteolin, quercetin, isorhamnetin, chlorogenic acid, rosmarinic acid, luteolin-4′-O-glucoside, luteolin-7-O-glucoside, hyperoside, luteolin-7-rutinoside, rutin, and isorhamnetin-3-O-rutinoside were purchased from Sigma-Aldrich (Saint Louis, MO, USA). During this research, purified deionized water was prepared using the Milli-Q^®^ (Millipore, Bedford, MA, USA) water purification system. All chemicals used were of analytical grade.

### 3.2. Plant Materials

*O. vulgare* herb was purchased from the University Pharmacy at the Lithuanian University of Health Sciences operating in Kaunas (Lithuania). Medical herbs were ground to a fine fraction powder using a mill (IKA^®^ A11 basic, Staufen, Germany). Loss on drying before all analysis was determined by drying about 1 g of powdered *O. vulgaris* herb material in a moisture analyzer (Precisa HA 300, Precisa Instruments AG, Dietikon, Switzerland) until complete evaporation of H_2_O and volatile compounds has been done, at a drying temperature of 105 °C. All data were recalculated for absolute dry weight (DW).

### 3.3. Preparation of Plant Extracts

An amount of 200 mL of distilled water was mixed with 50 g of dried *O. vulgare* herb powder; the mixture was allowed to expand for 3 h at room temperature. After that, the combined mixture was continually stirred at +55 °C in a magnetic stirrer. The extract was filtered and centrifuged. The extract was clear, uniform, and slightly yellow.

### 3.4. Preparation of Medusomyces Gisevii Fremented Media

Media of *Medusomyces gisevii* were prepared by adding 5 g of *O. vulgare* herb powder to 200 mL distilled hot water and stirring for 30 min at the temperature of +40 °C. After half an hour, 10 g of sugar was poured in; the mixture was stirred for another half hour. The nutrient-satisfied media solution was filtered and centrifuged until it was very clear. Then, it was left to cool down to room temperature. A thin layer of mushroom was obtained from the Laboratory of Biochemistry and Technology, Institute of Horticulture, Lithuania, and poured into a clean glass jar along with 100 mL of former media. Jar was cover with paper towel with rubber band and placed in dark and warm room with regulated temperature of about +20–30 °C for 6–8 days.

### 3.5. Green Synthesis of Silver Nanoparticles

About 0.03 g of AgNO_3_ was dissolved by vigorously mixing 50 mL of *O. vulgare* herb aqueous extracts with 10 mg of distilled water while constantly stirring at +50 °C for three hours. The obtained solution was incubated at room temperature for twenty-four hours in the dark. During synthesis, major change in color was observed: the mild yellow color changed to brown (dark) in reaction mixture, which confirmed the presence of silver nanoparticles.

### 3.6. Determination of Total Phenolic Content and Antioxidant Activity

All the spectrophotometric measurements were carried out with a UV-Vis spectrophotometer (Jinan Hanon Instrument Co., Ltd., Jinan, China). The total phenolic content (mg GAE/g) in the ethanol extracts of *O. vulgare* herb was determined by the Folin–Ciocâlteu method.

ABTS•+ Radical Cation Decolorization Assay: An ABTS•+ radical cation decolorization assay was applied according to the methodology described by Re et al. [36]. A volume of 3 mL of ABTS•+ solution (absorbance 0.800 ± 0.02) was mixed with 10 μL of the extract of oregano herb. A decrease in absorbance was measured at a wavelength of 734 nm after keeping the samples for 30 min in the dark.

CUPRAC Assay: CUPRAC solution included copper (II) chloride (0.01 M in water), ammonium acetate buffer solution (0.001 M, pH = 7), and neocuproine (0.0075 M in ethanol) (ratio 1:1:1). During the evaluation, 3 mL of CUPRAC reagent was mixed with 10 µL of extracts. An increase in absorbance was recorded after 30 min at a wavelength of 450 nm.

FRAP Assay: The ferric reducing antioxidant power (FRAP) assay was carried out as described in [28]. The working FRAP solution included TPTZ (0.01 M dissolved in 0.04 M HCl), FeCl_3_ × 6H_2_O (0.02 M in water), and acetate buffer (0.3 M, pH 3.6) at the ratio of 1:1:10. A volume of 3 mL of a freshly prepared FRAP reagent was mixed with 10 μL of the oregano herb extract. An increase in absorbance was recorded after 30 min at a wavelength of 593 nm.

The antioxidant activity in vitro of tested extracts was calculated from the Trolox calibration curve and expressed as mmol Trolox equivalent (TE) per gram.

### 3.7. Evaluation of Phenolic Compounds in Oregano Herb Samples Using the UHPLC-ESI-MS/MS Technique

The variability in phenolic compounds’ qualitative and quantitative content in oregano herb samples was evaluated by applying ultra-high performance liquid chromatography (UHPLC) mass spectrometry, using a technique described and validated in reference. The analysis of the qualitative and quantitative content of phenolic compounds in the samples of the oregano herb extracts was carried out using a liquid chromatography system, Waters ACQUITY UPLC^®^ H–Class (Waters, Milford, MA, USA), with the Xevo TQD tandem quadrupole mass detector (Waters, Milford, MA, USA). Sorting out of the compounds was performed using a YMC Triart C18 (100 Å, 100 × 2.0 mm; particle size of 1.9 μm) column (YMC, Kyoto, Japan) with a pre-column. The mass spectrometry parameters for the analysis of phenolic compounds are presented in Table 5.

Column temperature was maintained at 40 °C. Gradient elution was performed with mobile phase consisting of 0.1% formic acid water solution (solvent A) and acetonitrile (solvent B) with flow rate set to 0.5 mL min^−1^. Linear gradient profile was applied as follows for solvent A: initially, 95% for 1 min; then, 70% over 4 min; 50% over 7 min; 95% over 2 min. Negative electrospray ionization was applied for analysis: capillary voltage, −2 kV; source temperature, 150 °C; desolvation temperature, 400 °C; desolvation gas flow, 700 L h^−1^; cone gas flow, 20 L h^−1^. Collision energy and cone voltage were optimized for each compound separately.

### 3.8. Morphological Characterization of Plant Extracts and AgNPs

The morphology of the surface and chemical composition of the AgNPs were examined using FEi Quanta 200 FEG SEM technology, which has an acceleration voltage of twenty kV and a wavelength of 1.2 nm (FEI, USA). The Bruker XFlash^®^ 4030 (FEI, USA), an X-ray dispersive detection device, signal processor, ESPRT, and controller data analysis software, composed the EDS system. By identifying chemical components in the sample’s vicinity and visualizing the distribution of each chemical element on the surface, the spectrometer enables both qualitative and quantitative assessment of the chemical composition of the specimen. Furthermore, the structural transformations and physical characteristics of the object under investigation were evaluated qualitatively with the help of this research. To ensure comprehensive results, at least three scans were carried out on the samples.

Tecnai G2 F20 X-TWIN TEM (FEI, USA) was used to determine the structure, size, and distribution of the size of the investigated nanoparticles. An accelerating voltage between 20 and 200 kV was applied to the Schottky-type emission electron source. The microscope had a resolution of 0.8–1.0 nm. We employed an 11 MPix ORIUS SC1000B (Gatan) CCD camera and an EDAX spectrometer equipped with an r-TEM detector; 0.25/0.102 nm was the point/line resolution.

### 3.9. Antimicrobial Activity of O. vulgare

*O. vulgare* herb extracts and AgNPs were used to investigate the antibacterial effects of Gram-negative and Gram-positive bacteria strains. The antibacterial effect of extract was evaluated using an agar-based diffusion assay. Sterilized cotton swabs were used to inject each pathogenic bacterial strain with a density suspension of (0.5) half a McFarland unit (~108 CFU mL) onto the surface of chilled Mueller–Hinton agar (Oxoid, UK). Samples were placed into 6 mm diameter wells that were blown out of the agar. The mean dimension of the inhibition zones was determined with the help of trials that were performed three times. The inhibitory zones’ diameter (mm) was used to calculate the antibacterial activity against the tested microorganisms. This experiment was conducted using Gram-positive bacteria *Staphylococcus aureus* (*S. aureus*), *beta-hemolytic streptococcus group b* (*ß-streptococcus*), *Staphylococcus epidermidis* (*S. epidermidis*), *Bacillus cereus* (*B. cereus*), *Enterococcus faecalis* (*E. faecalis*); *Gram-negative bacteria: Escherichia coli* (*E. coli*), *Klebsiella pneumoniae* (*K. pneumoniae*), *Pseudomonas aeruginosa* (*P. aeruginosa*), *Proteus vulgaris* (*P. vulgaris*), and the fungi *Candida albicans* (*C. albicans*).

### 3.10. Statistical Analysis

Each experiment was carried out three times, and an average ± SD (standard deviation) was used to express all the data. GraphPad Prism 8 (GraphPad, San Diego, CA, USA) was used to calculate Tukey’s HSD tests. One-way ANOVA was used to evaluate the statistically significant variations (*p* < 0.05).

## 4. Conclusions

AgNPs have wide applications in various fields of cosmetics, biomedicine, renewable energy, healthcare, agriculture, sensors, water treatment, etc. The major aim of this study was to synthesize AgNPs using fermented *O*. *vulgare* herb extract and enhance their antimicrobial and antioxidant activity, along with the study of their photochemical composition. Biosynthesized particles were spherical in shape and demonstrated maximum antioxidant activity due to their bioactive molecules. During characterization of the samples, SEM indicated that their size was about <50 nm and that the NPs had a spherical shape of with a high degree of antibacterial activity. The antibacterial activity of this plant extract shows effectiveness in decreasing microbial growth. The highest level of activity was observed against *Staphylococcus aureus*, with an inhibition zone of 1.40–11.54 mm overall. The results of our investigation of antioxidant activity revealed that fermented *O. vulgare* herb extract showed a stronger reductive activity, as evaluated by cupric reducing antioxidant capacity (CUPRAC), ferric reducing antioxidant power (FRAP), and 2,2′-azino-bis (3-ethylbenzothiazoline-6-sulfonic acid) (ABTS•+). All extracts contain flavonoids, hydroxycinnamic acid, and derivatives of phenolic acid, which provides antioxidant and antimicrobial activity. Despite the various advantages of the material, the literature still lacks a description of the specific mechanism for the synthesis of AgNPs using fermented plant extracts, especially with a symbiotic culture of *Medusomyces gisevii*.

## Figures and Tables

**Table 1:** Variability in the content of phenolic compounds in pure OrV, OrV-AgNPs, and OrV-F-AgNPs samples was evaluated via the UPLC-ESI-MS/MS technique.

Compound	OrV, µg/g	OrV-AgNPs, µg/g	OrV-F-AgNPs, µg/g
Luteolin	16.88 ± 1.44	51.38 ± 0.69	19.94 ± 0.40
Luteolin-4′-*O*-glucoside	12.59 ± 0.47	21.32 ± 1.79	47.72 ± 3.24
Luteolin-7-rutinoside	11.66 ± 0.67	19.64 ± 1.61	38.17 ± 1.91
Chlorogenic acid	24.54 ± 1.52	22.78 ± 0.50	43.55 ± 4.05
Quercetin	48.00 ± 4.28	28.47 ± 0.58	37.95 ± 2.63
Isorhamnetin-3-*O*-rutinoside	76.98 ± 5.64	71.63 ± 6.86	27.29 ± 1.39
*p*–Coumaric acid	55.47 ± 4.57	46.47 ± 4.12	35.6 ± 1.96
Galangin	45.12 ± 3.12	57.29 ± 5.16	54.19 ± 4.29
Isorhamnetin	74.11 ± 4.54	12.78 ± 0.52	13.33 ± 0.97
Apigenin	48.44 ± 3.06	16.56 ± 0.64	87.78 ± 8.40
Rutin	14.22 ± 0.67	68.89 ± 2.20	13.83 ± 1.08
Hyperoside	32.22 ± 1.75	37.16 ± 1.95	96.18 ± 1.86
Naringenin	47.78 ± 4.21	28.67 ± 1.62	24.78 ± 2.17
Rosmarinic acid	3793 ± 255.3	256.3 ± 11.6	3272 ± 213.8
Luteolin-7-*O*-glucoside	22.47 ± 1.14	14.37 ± 1.22	14.35 ± 0.64
Total	4323 ± 285.6	753.68 ± 66.16	3827.17 ± 99.88

Note: OrV—*O. vulgare*, OrV-AgNPs—*O. vulgare* with silver nanoparticles, OrV-F-AgNPs—fermented silver nanoparticles in *O. vulgare* extract.

**Table 2:** Phytochemical analysis of plant extracts and biosynthesized AgNPs.

Compound Name	OrV	OrV-AgNPs	OrV-F-AgNPs
The total content of phenolic compounds, mg GAE/g DW	210.17 ± 6.48	206.00 ± 0.59	257.25 ± 8.25
The total amount of proanthocyanidins, mg EE/g DW	102.1 ± 0.03	87.5 ± 0.01	145.00 ± 0.02
The total amount of hydroxycinnamic acid derivatives, mg CAE/g DW	2.78 ± 0.02	1.32 ± 0.04	2.86 ± 0.01
The total amount of phenolic compounds, mg GAE/g DW	3.05 ± 0.01	1.90 ± 0.02	2.56 ± 0.01
The total amount of flavonoids, mg RE/g DW	0.67 ± 0.01	0.17 ± 0.01	0.55 ± 0.01

Note: OrV—*O. vulgare*, OrV-AgNPs—*O. vulgare* with silver nanoparticles, OrV-F-AgNPs—fermented silver nanoparticles in *O. vulgare* extract.

**Table 3:** Antioxidant activity in vitro of tested *O*. *vulgare* herb extracts and biosynthesized AgNPs (*p* > 0.05).

Assay	OrV	OrV-AgNPs	OrV-F-AgNPs
ABTS, mmol TE/g	0.74 ± 0.002	0.65 ± 0.006	0.59 ± 0.023
FRAP, mmol TE/g	1.11 ± 0.04	1.02 ± 0.08	0.95 ± 0.04
CUPRAC, mmol TE/g	1.59 ± 0.003	1.54 ± 0.007	1.45 ± 0.048

Note: OrV—*O. vulgare*, OrV-AgNPs—*O. vulgare* with silver nanoparticles, OrV-F-AgNPs—fermented silver nanoparticles in *O. vulgare* extract, ABTS—(2,2′-azino-bis(3-ethylbenzothiazoline-6-sulfonic acid)), FRAP—ferric reducing antioxidant power, CUPRAC—cupric reducing antioxidant capacity.

**Table 4:** Inhibition zones of the plant extracts and AgNPs against Gram-positive and Gram-negative bacteria strains (*p* > 0.05).

Cultures of Microorganisms	OrV	OrV-AgNPs	OrV-F-AgNPs
	Inhibition zone (mm)		
*Staphylococcus aureus*	1.40 ± 0.12	10.30 ± 0.04	11.54 ± 0.10
*ß-streptococcus*	0.40 ± 0.10	9.40 ± 0.05	10.85 ± 0.05
*Staphylococcus epidermidis*	0.00 ± 0.00	9.05 ± 0.15	10.95 ± 0.40
*Escherichia coli*	0.00 ± 0.00	5.50 ± 0.20	5.80 ± 0.03
*Klebsiella pneumoniae*	0.00 ± 0.00	4.20 ± 0.01	5.15 ± 0.01
*Pseudomonas aeruginosa*	0.00 ± 0.00	3.85 ± 0.10	4.02 ± 0.10
*Proteus vulgaris*	0.00 ± 0.00	4.10 ± 0.25	5.17 ± 0.16
*Bacillus cereus*	0.00 ± 0.00	5.85 ± 0.17	7.00 ± 0.08
*Enterococcus faecalis*	0.00 ± 0.00	6.85 ± 0.05	8.50 ± 0.05
*Candida albicans*	0.00 ± 0.00	4.89 ± 0.10	6.25 ± 0.10

Note: OrV—*O. vulgare*, OrV-AgNPs—*O. vulgare* with silver nanoparticles, OrV-F-AgNPs—fermented silver nanoparticles in *O. vulgare* extract.

**Table 5:** Mass spectrometry parameters for the analysis of phenolic compounds.

Compound	Parent Ion (*m*/*z*)	Daughter Ion (*m*/*z*)	Cone Voltage, V	Collision Energy, eV
*p*–Coumaric acid	163	93	28	22
Galangin	269	171	50	30
Apigenin	269	117	54	36
Naringenin	271	151	46	18
Luteolin	285	133	58	36
Quercetin	301	151	48	18
Isorhamnetin	315	300	44	22
Chlorogenic acid	353	191	32	14
Rosmarinic acid	359	161	36	16
Luteolin-4′-*O*-glucoside	447	285	36	16
Luteolin-7-*O*-glucoside	447	285	66	26
Hyperoside	463	300	50	26
Luteolin-7-rutinoside	593	285	82	36
Rutin	609	300	70	38
Isorhamnetin-3-*O*-rutinoside	623	315	70	32

## Data Availability

All data generated during this study are included in this article.
